# Identification of a Major Phosphopeptide in Human Tristetraprolin by Phosphopeptide Mapping and Mass Spectrometry

**DOI:** 10.1371/journal.pone.0100977

**Published:** 2014-07-10

**Authors:** Heping Cao, Leesa J. Deterding, Perry J. Blackshear

**Affiliations:** 1 U. S. Department of Agriculture, Agricultural Research Service, Southern Regional Research Center, New Orleans, Louisiana, United States of America; 2 Laboratory of Structural Biology, National Institute of Environmental Health Sciences, National Institutes of Health, Research Triangle Park, North Carolina, United States of America; 3 Laboratory of Signal Transduction, National Institute of Environmental Health Sciences, National Institutes of Health, Research Triangle Park, North Carolina, United States of America and Departments of Biochemistry and Medicine, Duke University Medical Center, Durham, North Carolina, United States of America; SRI International, United States of America

## Abstract

Tristetraprolin/zinc finger protein 36 (TTP/ZFP36) binds and destabilizes some pro-inflammatory cytokine mRNAs. TTP-deficient mice develop a profound inflammatory syndrome due to excessive production of pro-inflammatory cytokines. TTP expression is induced by various factors including insulin and extracts from cinnamon and green tea. TTP is highly phosphorylated *in vivo* and is a substrate for several protein kinases. Multiple phosphorylation sites are identified in human TTP, but it is difficult to assign major vs. minor phosphorylation sites. This study aimed to generate additional information on TTP phosphorylation using phosphopeptide mapping and mass spectrometry (MS). Wild-type and site-directed mutant TTP proteins were expressed in transfected human cells followed by *in vivo* radiolabeling with [^32^P]-orthophosphate. Histidine-tagged TTP proteins were purified with Ni-NTA affinity beads and digested with trypsin and lysyl endopeptidase. The digested peptides were separated by C_18_ column with high performance liquid chromatography. Wild-type and all mutant TTP proteins were localized in the cytosol, phosphorylated extensively *in vivo* and capable of binding to ARE-containing RNA probes. Mutant TTP with S^90^ and S^93^ mutations resulted in the disappearance of a major phosphopeptide peak. Mutant TTP with an S^197^ mutation resulted in another major phosphopeptide peak being eluted earlier than the wild-type. Additional mutations at S^186^, S^296^ and T^271^ exhibited little effect on phosphopeptide profiles. MS analysis identified the peptide that was missing in the S^90^ and S^93^ mutant protein as LGPELSPSPTSPTATSTTPSR (corresponding to amino acid residues 83–103 of human TTP). MS also identified a major phosphopeptide associated with the first zinc-finger region. These analyses suggest that the tryptic peptide containing S^90^ and S^93^ is a major phosphopeptide in human TTP.

## Introduction

Tristetraprolin (TTP) is the prototypic member of a small family of tandem C_8_C_5_C_3_H zinc finger proteins (ZFP). Similar tandem C_8_C_5_C_3_H zinc finger sequences have been found in many species, ranging from human through yeasts and plants [1]–[3]. The TTP protein family consists of three members common to mammals (ZFP36 or TTP, ZFP36L1 or TIS11B and ZFP36L2 or TIS11D) and a fourth member in mouse and rat but not in humans (ZFP36L3) [1], [4]. TTP family proteins bind to AU-rich elements (ARE) within single stranded RNAs [5]–[11] and promote the deadenylation and subsequent destruction of those transcripts [12], [13]. TTP-deficiency in knockout mice causes a severe inflammatory syndrome with erosive arthritis, autoimmunity and myeloid hyperplasia [10], [14]. This is largely due to excessive production of pro-inflammatory cytokines including tumor necrosis factor alpha (TNFα) and granulocyte-macrophage colony-stimulating factor, whose mRNAs are direct targets of TTP but are stabilized in TTP knockout mice cells [8], [12], [15]. TTP is therefore regarded as an anti-inflammatory protein.

TTP protein expression is induced in various cell types by a number of factors including insulin [16], lipopolysaccharide (LPS) [17] and cinnamon polyphenolic extract [12], [18]–[20]. TTP is highly phosphorylated in intact cells and in cell-free systems [9], [12], [20]–[23]. TTP is a substrate for a number of protein kinases such as p42 mitogen-activated protein (MAP) kinase (ERK2) [6], [7], [23], p38 MAP kinase [6], [7], [9], [24], c-Jun N-terminal kinase (JNK) [6], MAP kinase-activated protein kinase 2 (MAPKAP kinase 2 or MK2) [25]–[28], glycogen synthase kinase-3β [29] and protein kinases A, B and C [29]. Mass spectrometry (MS) and site-directed mutagenesis have identified a number of phosphorylation sites in human and mouse TTP (hTTP and mTTP) [3], [21], [23], [25]. However, it is puzzling that mutant TTP with extensive mutations is still phosphorylated extensively *in vivo* [21], [30]. Recent studies have shown that mutant hTTP with some phosphorylation sites mutated could be a potent inhibitor of malignant glioma cell growth [31]. Therefore, it is important to understand the structure-function relationships of TTP phosphorylation.

Several approaches have been used to identify TTP phosphorylation sites including *in vivo* labeling, site-directed mutagenesis, mass spectrometry and computational analysis. One major problem is that the major phosphorylation sites identified by mass spectrometry are not necessarily in agreement among different laboratories [3], [21], [25]. For example, S^52^, S^178^ and S^220^ of mTTP (corresponding to S^60^, S^186^ and S^228^ of hTTP) are the major phosphorylation sites in mTTP, but the human equivalent S^60^ is not identified as a major site in hTTP [21], [25], [28], [32]. Another example is that S^105^ and S^316^ of mTTP are phosphorylated in intact cells [25] but the equivalent sites at S^113^ or S^323^ of hTTP are not confirmed in transfected human cells [21].

In this study, we extended our investigation on the identification of potential phosphorylation sites by phosphopeptide mapping, site-directed mutagenesis and mass spectrometry. Our results demonstrated that mutations at both S^90^ and S^93^ in hTTP resulted in the disappearance of a major phosphopeptide peak in the HPLC chromatogram. The missing phosphopeptide identified by MS contained both S^90^ and S^93^, suggesting that the tryptic peptide is a major phosphopeptide in hTTP from transfected human cells.

## Results

### Expression of TTP Proteins in Transfected Human Cells

**Figure 1:**
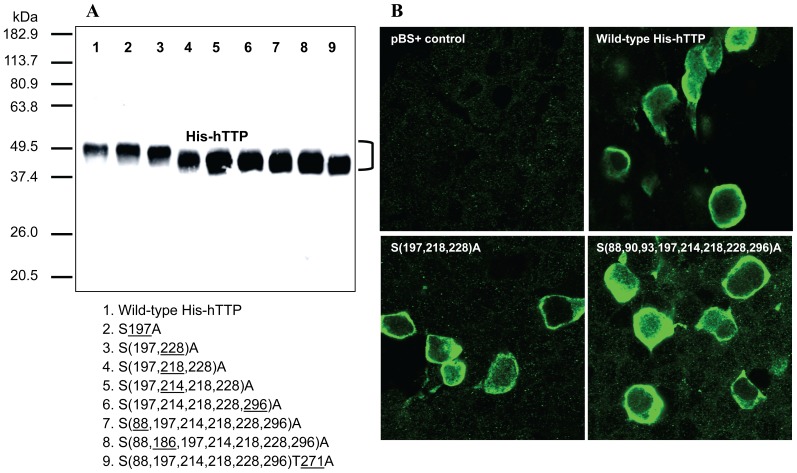
Expression and localization of the wild-type and mutant hTTP in transfected human cells. (A) Immunoblotting. HEK293 cells were transfected with pHis-hTTP plasmids. Proteins in the soluble extracts (10,000*g*, 10 µg/lane) were separated by SDS-PAGE (10% Tris-glycine gel) and transferred onto nitrocellulose membrane. The membrane was incubated in anti-MBP-hTTP serum (1∶10,000 dilution, 2 h) followed by secondary antibodies (1∶10,000 dilution, 1 h). The blot was incubated in Super Signal for 5 min and exposed to X-ray film for 5 sec. The underlined numbers in the plasmids 1–9 below the gel represent the sites of serine/threonine residues mutated to alanine residues in addition to the mutations of hTTP in the preceding plasmid. (B) Immunostaining. HEK293 cells were transfected with pBS+ control plasmid and pHis-hTTP plasmids encoding wild-type His-hTTP and mutant His-hTTP with S(214,218,228)A and S(88, 90, 93, 197, 214, 218, 228, 296)A mutations. The cells were stained with anti-MBP-hTTP antibodies (1∶5,000 dilution, overnight) and labeled with goat anti-rabbit Alexa Fluor 488 (1∶1,000 dilution, 1 h). Immunofluorescence was recorded by confocal microscopy.

HEK293 cells were transfected with pHis-hTTP plasmids encoding wild-type and mutant proteins with serine and threonine to alanine mutation(s) in hTTP. Immunoblotting showed that all His-hTTP proteins were expressed in the transfected cells (Figure 1A). Immunostaining with TTP antibodies showed that endogenous TTP was undetectable in HEK293 cells transfected only with the pBS+ carrier plasmid (Figure 1B). Wild-type TTP was overexpressed and mainly localized in the cytosol of HEK293 cells transfected with wild-type pHis-hTTP plasmid (Figure 1B). Mutant TTP proteins were also expressed and primarily localized in the cytosol of transfected HEK293 cells in patterns similar to those of the wild-type TTP (Figure 1B).

### Multiple Distinctive Species of TTP Proteins from Transfected Human Cells

**Figure 2:**
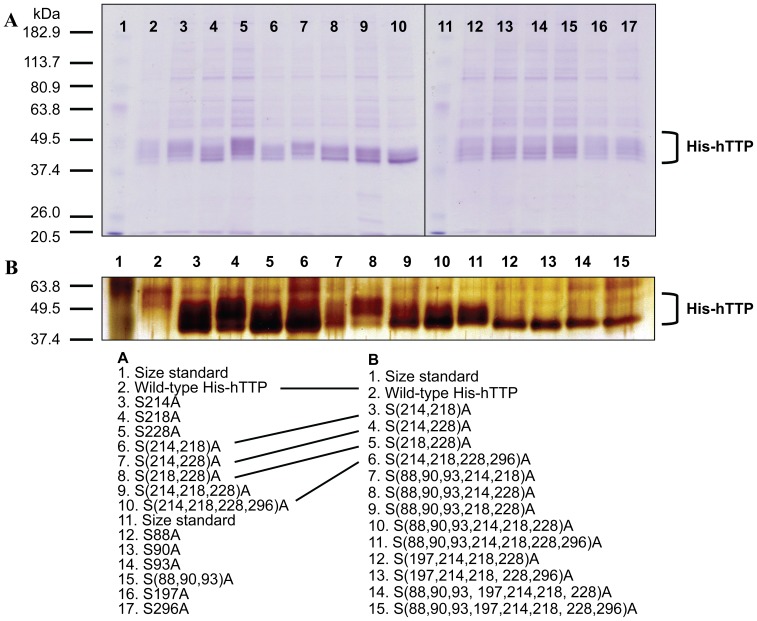
Multiple distinct bands of the wild-type and mutant hTTP purified from transfected human cells. HEK293 cells were transfected with the selected plasmids. Proteins in the soluble extracts were bound to Ni-NTA beads and eluted with 100 mM imidazole solution. Proteins were separated by SDS-PAGE (10% Tris-glycine gel). (A) Coomassie brilliant blue staining (20 µL of protein). The gel was fixed with 10% acetic acid/25% isopropanol, stained with 0.006% Coomassie brilliant blue in 10% acetic acid overnight and destained with 10% acetic acid. (B) Silver staining (2 µL of protein). The identical proteins used in both panels are linked with a line.

Wild-type and a number of mutant His-hTTP proteins were purified by Ni-NTA beads and eluted with an imidazole solution. The purified proteins were separated by SDS-PAGE and stained with Coomassie brilliant blue (CBB) and silver reagents for observing the electrophoretic mobility of the proteins. CBB staining is less sensitive so that minor size differences between TTP protein bands could be seen on the gel. Silver staining is more sensitive, which could easily obscure the neighboring TTP bands, resulting in a smear or fat band instead of distinctive bands. CBB staining showed that the wild-type and mutant hTTP proteins with 1–4 mutations exhibited multiple distinctive bands on protein gels (Figure 2A). Silver staining showed that the same wild-type and mutant hTTP proteins exhibited a fat band on the protein gel (Lanes 2–6 in Figure 2B were identical to lanes 2, 6, 7, 8, and 10 in Figure 2A). Mutations at additional sites in hTTP resulted in apparently single band or sharp bands on the protein gel (Figure 2B, lanes 12–15). These results suggest that mutations at multiple potential phosphorylation sites can have a large effect on the electrophoretic mobility of the human TTP protein.

### Phosphopeptide Mapping of Wild-type TTP

**Figure 3:**
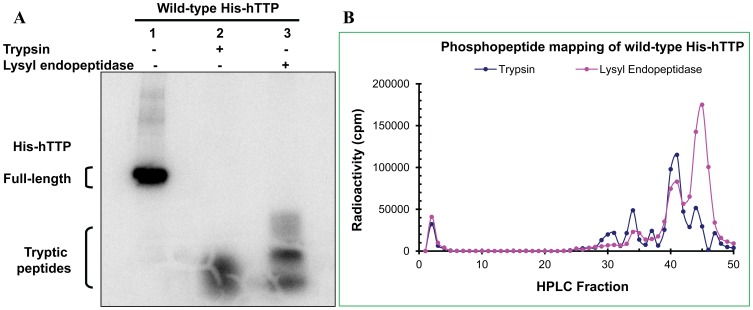
Phosphopeptide mapping of the wild-type hTTP protein from transfected human cells. HEK293 cells were transfected with the wild-type pHis-hTTP plasmid followed by *in vivo* radiolabeling with [^32^P]-orthophosphate. Proteins in the soluble extracts were bound to Ni-NTA beads. The bound proteins were eluted with 250 mM imidazole solution. Proteins were digested overnight with trypsin and lysyl endopeptidase. (A) Autoradiography. The undigested protein and digested peptides were separated by SDS-PAGE (4–20% Tris-glycine gel). The gel was dried and exposed to X-ray film. (B) HPLC separation. The digested peptides were separated by reverse phase HPLC and eluted from the column. The radioactivity of every fraction was counted and plotted.

Previous *in vivo* labeling studies showed that TTP was highly phosphorylated [21]. To further investigate TTP phosphorylation in the cells, we labeled HEK293 cells with [^32^P]-orthophosphate following transfection with the wild-type plasmid pHis-hTTP. The wild-type protein was purified from the 10,000*g* supernatant by Ni-NTA affinity beads. SDS-PAGE followed by autoradiography showed that hTTP was essentially the only phosphoprotein purified by this procedure (Figure 3A, lane 1). The purified proteins were completed digested for extended time into smaller fragments by trypsin and lysyl endopeptidase (Figure 3A, lanes 2–3). These peptides were separated by reverse-phase HPLC and radioactivity in each fraction was counted. Phosphopeptide mapping showed that several radioactive peaks were present in the trypsin and lysyl endopeptidase digests, and the first small peak of radioactivity was washed off the column (Figure 3B). These results are in agreement with a previous report that hTTP is phosphorylated at multiple sites in intact cells [21].

### Phosphopeptide Mapping of TTP with Mutations at S^214^, S^218^ and S^228^

**Figure 4:**
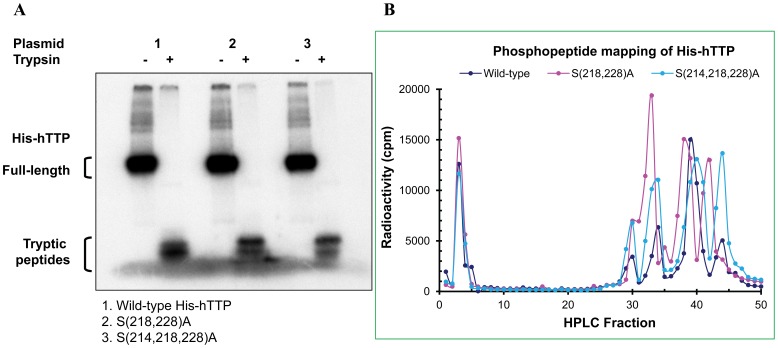
Phosphopeptide mapping of the wild-type and mutant hTTP protein with S^214^, S^218^ and S^228^ mutations from transfected human cells. HEK293 cells were transfected with the wild-type and mutant plasmids with S(218,228)A and S(214,218,228)A mutations followed by *in vivo* radiolabeling with [^32^P]orthophosphate. Proteins in the soluble extracts were bound to Ni-NTA beads and eluted with 250 mM imidazole solution. Proteins were digested overnight with trypsin followed by HPLC separation. (A) Autoradiography. The undigested protein and digested peptides were separated by SDS-PAGE (8–16% Tris-glycine gel). The gel was dried and exposed to X-ray film. (B) HPLC separation. The trypsin-digested peptides were separated by reverse phase HPLC and eluted from the column. The radioactivity of every fraction was counted and plotted.

S^218^ and S^228^ are two of the major phosphorylation sites in hTTP [21]. To investigate if TTP with these two mutations lacks any phosphopeptide, we labeled HEK293 cells transfected with plasmids encoding hTTP with double mutations at the S^218^ and S^228^ sites and hTTP with triple mutations at the S^214^, S^218^ and S^228^ sites. The proteins were purified by Ni-NTA beads and digested with trypsin. Autoradiography showed that the full-length wild-type hTTP and the two hTTP mutant proteins were digested by trypsin, resulting in smaller size bands on SDS-PAGE (Figure 4A). Surprisingly, phosphopeptide profiles showed that the wild-type and the mutant hTTP proteins contained the same numbers of phosphopeptide peaks, although there were some minor differences in retention time of phosphopeptides among the three proteins (Figure 4B).

### Phosphopeptide Mapping of TTP with More Mutations

**Figure 5:**
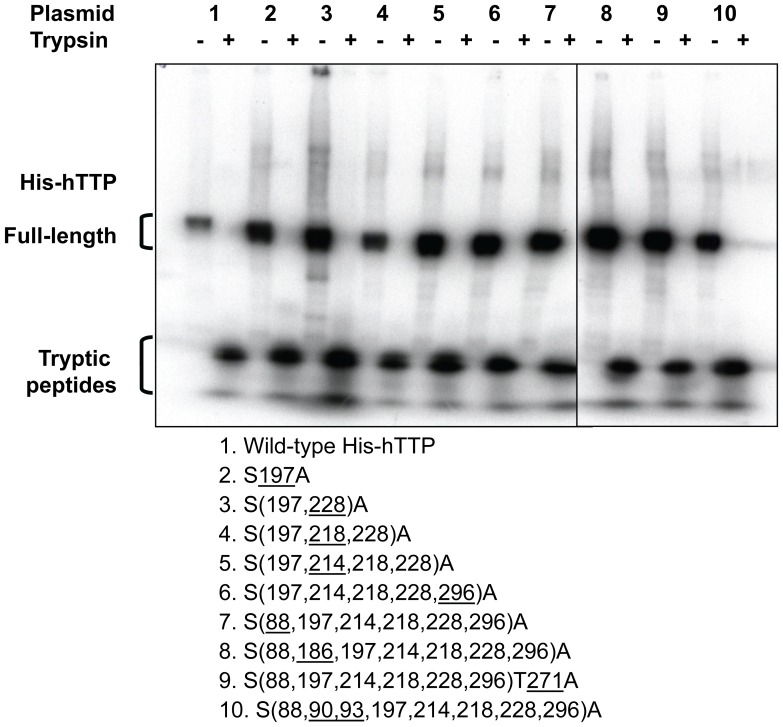
Trypsin digestion of the wild-type and mutant hTTP proteins from transfected human cells. HEK293 cells were transfected with the wild-type and 9 mutant plasmids followed by *in vivo* radiolabeling with [^32^P]-orthophosphate. Proteins in the soluble extracts were bound to Ni-NTA beads and eluted with imidazole solution. Proteins were digested with trypsin. The undigested protein and digested peptides were separated by SDS-PAGE (8–16% Tris-glycine gel). The gel was dried and exposed to X-ray film. The underlined numbers in the plasmids 1–10 below the gel represent the sites of serine/threonine residues mutated to alanine residues in addition to the mutations of hTTP in the preceding plasmid.

HEK293 cells were transfected with pHis-hTTP plasmids encoding wild-type and nine mutant hTTP proteins. HEK293 cells were then labeled with [^32^P]-orthophosphate. The wild-type and mutant His-hTTP proteins were purified from the 10,000*g* supernatant by Ni-NTA affinity beads. Autoradiography showed that the proteins appeared to be labeled to similar extents, despite their extensive mutations (Figure 5). The radiolabeled proteins were digested to completion with TPCK-treated trypsin, as judged by SDS-PAGE and autoradiography (Figure 5).

**Figure 6:**
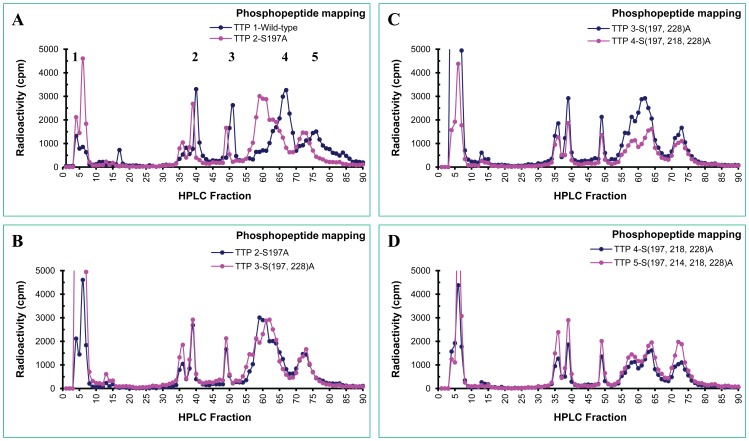
Phosphopeptide mapping of the wild-type and mutant hTTP proteins from transfected human cells. Wild-type and mutant hTTP proteins were labeled with [^32^P]-orthophosphate in HEK293 cells. His-hTTP was purified and digested by trypsin as described in Figure 5 legend. The trypsin-digested peptides were separated by reverse phase HPLC and eluted from the column at 0.5 mL/min with 20-bed volume of a linear gradient. The fractions were collected at 0.25 mL/well in 96-well plates. The radioactivity of every fraction was counted and plotted. The radioactivity in the plots from the wild-type proteins was five times of the actual radioactivity for visual comparisons of its phosphopeptides to the mutant ones because of the low recovery of phosphopeptides of the wild-type protein in HPLC fractions (Table 1). The radioactivity in the plots from the mutant proteins was the actual radioactivity. The phosphopeptide profiles in each pair of hTTP proteins are (A) Wild-type vs. S197A, (B) S197A vs. S(197, 228)A, (C) S(197,228)A vs. S(197,218,228)A, and (D) S(197,218,228)A vs. S(197,214,218,228)A.

**Figure 7:**
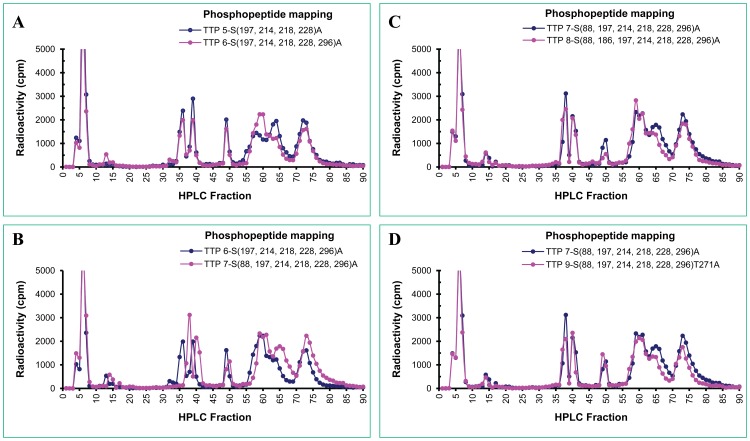
Phosphopeptide mapping of mutant hTTP proteins from transfected human cells. The methods for generating the phosphopeptide mapping profiles were identical to those described in Figure 6 legend. The phosphopeptide profiles in each pair of hTTP proteins are (A) S(197,214,218,228)A vs. S(197,214,218,228,296)A, (B) S(197,214,218,228,296)A vs. S(88,197,214,218,228,296)A, (C) S(88,197,214,218,228,296)A vs. S(88,186,197,214,218,228,296)A, and (D) S(88,197,214,218,228,296)A vs. S(88,197,214,218,228,296)T271A.

**Figure 8:**
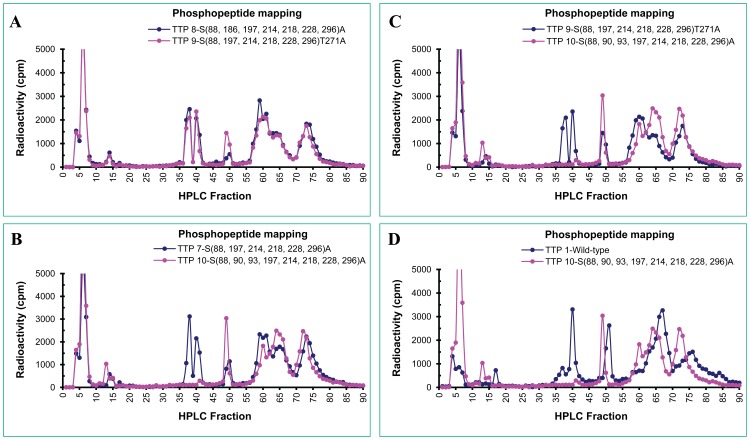
Phosphopeptide mapping of the wild-type and mutant hTTP proteins from transfected human cells. The methods for generating the phosphopeptide mapping profiles were identical to those described in Figure 6 legend. The phosphopeptide profiles in each pair of hTTP proteins are (A) S(88,186,197,214,218,228,296)A vs. S(88,197,214,218,228,296)T271A, (B) S(88,197,214,218,228,296)A vs. S(88,90,93,197,214,218,228,296)A, (C) S(88,197,214,218,228,296)T271A vs. S(88,90,93,197,214,218,228,296)A, and (D) Wild-type vs. S(88,90,93,197,214,218,228,296)A.

The digested peptides were separated by reverse-phase HPLC through a C_18_ column and the radioactivity in each fraction was counted. The phosphopeptides from mutant hTTP contained more radioactivity than those from the wild-type hTTP (Table 1). The selected profiles of phosphopeptide mapping comparisons are shown in Figures 6–8. A comparison of phosphopeptide maps between wild-type and S197A mutant hTTP is shown in Figure 6A. The overall phosphopeptide maps were similar between these two proteins. The most striking difference between these two profiles was that the phosphopeptide peaks of the mutant protein were eluted earlier than those of the wild-type protein.

**Table 1:** HPLC recovery of radioactivity.

Plasmid no.	Construct(pHis-hTTP)	Radioactivity beforeseparation (cpm)	Radioactivity in thepellet (cpm)	Radioactivity recovered in theHPLC fractions (cpm)	HPLCrecovery (%)^1^	Totalrecovery (%)
1	Wild-type	42341	955	12455	30.1	31.7
2	S197A^2^	128820	2010	58776	46.3	47.2
3	S(197,228)A	192986	9872	87160	47.6	50.3
4	S(197,228,218)A	80527	2916	42395	54.6	56.3
5	S(197,228,218,214)A	124994	5617	57760	48.4	50.7
6	S(197,228,218,214,296)A	123763	4042	49752	41.6	43.5
7	S(197,228,218,214,296,88)A	147327	4000	61284	42.8	44.3
8	S(197,228,218,214,286,88,186)A	150917	8920	56555	39.8	43.4
9	S(197,228,218,214,296,88)T271A	114453	3318	51259	46.1	47.7
10	S(197,228,218,214,296,88,90,93)A	145495	3914	58768	41.5	43.1

1 The recovery of radioactivity of HPLC fractions from every mutant hTTP protein was higher than that from the wild-type protein.

2 The underlined numbers in the plasmids 1–10 below represent the sites of serine/threonine residues mutated to alanine residues in addition to the mutations of hTTP in the preceding plasmid.

The elution profiles of phosphopeptides of hTTP with an alanine mutation at S^197^ plus additional mutations at S^228^ (Figure 6B), S^(218,228)^ (Figure 6C), S^(214,218,228)^ (Figure 6D), or S^(214,218,228, 296)^ (Figure 7A) were similar to that of hTTP with the only mutation at S^197^ (Figure 6B). Additional mutation at S^88^ in additional to S^(197, 214,218,228, 296)^ clearly changed the elution pattern by increasing the retention time of the phosphopeptides (Figure 7B), but more mutations at S^186^ (Figure 7C) or T^271^ (Figure 7D and Figure 8A) did not exhibit significant effects on the phosphopeptide elution patterns.

The major effects of mutations on phosphopeptide mapping were observed when hTTP was mutated at S^(90.93)^. Two phosphopeptide peaks in fractions 37–43 disappeared in hTTP with these two mutations in addition to those mutations at S^(88, 197, 214,218,228, 296)^ (Figure 8B) or S^(88, 197, 214,218,228, 296)T271^ (Figure 8C). The two missing peaks in fractions 37–43 from hTTP containing S^90^ and S^93^ mutations corresponded to one major and one minor peak of radioactivity from the wild-type protein (Figure 8D).

### MALDI-MS Analysis of Phosphopeptides of TTP

**Figure 9:**
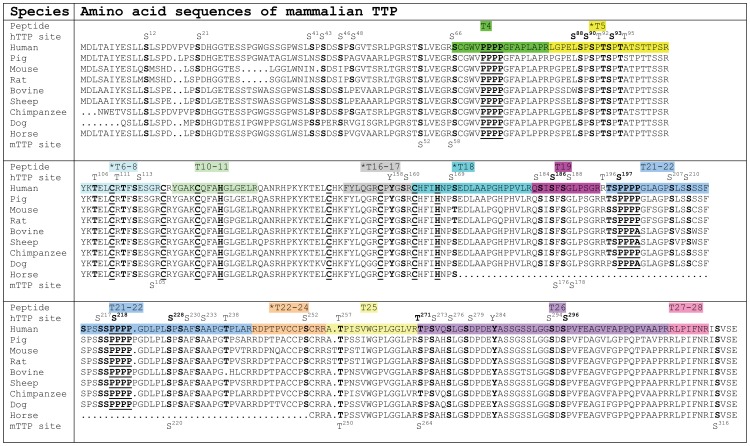
Amino acid sequence alignment of TTP from mammalian species with the major phosphopeptides identified in human and mouse TTP. The amino acid sequences used in the alignment include human (NP_003398) [40], mouse (NP_035886) [41], rat (P47973) [42], bovine (P53781) [43], sheep (AY462109) [44], pig (AJ943797, CB288050, CB286240, DY416794 and DY419026) [45], horse (CD536573 and CD536523), chimpanzee (CR555169 and XM_001136016) and dog (AAEX01054372.1 and AAEX01054371.1) [46]. The sequences were aligned with the PILEUP program from GCG. 180 amino acid residues in chimpanzee TTP N-terminal are not shown in the alignment: The gaps in the horse TTP sequence represent incomplete sequence and are probably highly similar to the other mammalian TTP sequences. The bold and underlined residues within the sequence alignment are the signature residues of TTP family proteins including the three repeats of four proline residues and the two repeats of the CX_8_CX_5_CX_3_H zinc-finger binding motifs. The bold but not underlined residues within the sequence alignment are the phosphorylation sites identified in hTTP and mTTP and the corresponding residues in other species. The conserved serine, threonine and tyrosine residues, which correspond to the phosphorylation sites in hTTP are indicated at the top of the sequence alignment. Those residues corresponding to the phosphorylation sites in mTTP are indicated at the bottom of the sequence alignment. The names of the tryptic peptides derived from hTTP observed by MALDI-MS are shown at the top of the alignment. The five tryptic peptides with “*” at the front of their names (T5, T6–8, T16–17, T18 and T22–24) indicate the major phosphopeptides identified in this study. The colors match the tryptic peptides and their corresponding sequences in the alignment (updated from Cao et al [3]).

To identify the phosphopeptides eluted from HPLC columns, we used MS methods to analyze the HPLC fractions with high levels of radioactivity from the wild-type His-hTTP (Figure 6A). The first major peak of radioactivity (Figure 6A, peak 1) washed off the column contained a phosphopeptide with the amino acid sequence CHFIHNPSEDLAAPGHPPVLR (Table 2). This peptide corresponds to amino acid residues 162–182 of hTTP (T18 in Figure 9). The underlined S^169^ site is the only phosphorylation site identified previously [21].

**Table 2:** MAIDI-MS analysis of HPLC fractions with major radioactivity.

Radioactivitypeak^1^	Observedmass (Da)^2^	Unmodifiedmass (Da)^3^	Differentialmass (Da)^4^	Trypticfragment	Region inhTTP	Amino acid sequence in hTTP(potential phosphorylation site underlined)	Corresponding to reportedphosphorylation site [21]
1	2387.24	2307.15	80.09	T18	162–182	(R) CHFIHNP**S**EDLAAPGHPPVLR (Q)	S^169^
2	2165.25	2084.05	81.2	T5	83–103	(R) LGPEL**S**P**S**P**TS**P**T**ATSTTPSR (Y)	S^88^, S^90^, T^92^, S^93^and T^95^
3	1650.98	1489.68	161.3	T22–24	243–255	(R) RDPTPVCCP**S**CRR (A)	S^252^
4	1757.12	1676.81	80.31	T6–8	104–117	(R) YK**T**ELCR**T**F**S**ESGR (C)	T^105^, T^111^ and S^113^
5	1526.92	1446.70	80.22	T16–17	150–161	(K) FYLQGRCP**Y**G**S**R (C)	Y^158^ and S^160^

1 The wild-type hTTP protein was purified from transfected human cells after *in vivo* radiolabeling with [^32^P]-orthophosphate. The protein was purified and digested by trypsin to completion. The phosphopeptides were identified by radioactivity peak on HPLC chromatogram (Figure 6A).

2 The observed peptide mass of [M+H] ion was obtained after phosphopeptides were sequenced by MAIDI-MS.

3 The unmodified peptide mass of [M+H] ion was obtained after theoretical digestion of His-hTTP with trypsin.

4 The differential mass was obtained by subtraction the unmodified ion mass from the observed ion pass. Phosphorylation results in a peptide ion with a +80 Da mass increase compared to the unmodified peptide for each phosphorylated Ser, Thr or Tyr residue (HPO_3_^−^ = 79.97 Da).

The second radioactivity peak (Figure 6A, peak 2) from the wild-type hTTP that disappeared in mutant hTTP with S^90^ and S^93^ mutations was determined to contain a phosphopeptide with the amino acid sequence LGPELSPSPTSPTATSTTPSR (Table 2). This peptide sequence corresponds to amino acid residues 83–103 of hTTP (T5 in Figure 9). This peptide contained five potential phosphorylation sites as previously identified by MS analyses (S^88^, S^90^, T^92^, S^93^and T^95^ sites) (Figure 9).

The third major peak of radioactivity (Figure 6A, peak 3) contained a phosphopeptide with the amino acid sequence RDPTPVCCPSCRR (Table 2) (corresponding to amino acid residues 243–255 of hTTP) (T22–24 in Figure 9). The S^252^ site is the only phosphorylation site identified previously [21]. This fraction might also contain a non-phosphorylated peptide with the amino acid sequence YGAKCQFAHGLGELR (corresponding to amino acid residues 120–134 of hTTP) (T10–11 in Figure 9) since this peptide has the same molecular mass.

The forth major peak of radioactivity (Figure 6A, peak 4) contained a phosphopeptide with the amino acid sequence YKTELCRTFSESGR (Table 2) (corresponding to amino acid residues 104–117 of hTTP) (T6–8 in Figure 9). It is notable that this peptide is located in the first C_8_C_5_C_3_H zinc-finger region with three potential phosphorylation sites at T^106^, T^111^ and S^113^ in hTTP (Figure 9).

The last major peak of radioactivity (Figure 6A, peak 5) contained a phosphopeptide with the amino acid sequence FYLQGRCPYGSR (Table 2) (corresponding to amino acid residues 150–161 of hTTP) (T16–17 in Figure 9). The Y^158^ and S^160^ sites are phosphorylation sites identified previously [21].

### RNA-binding Activity of Wild-type and Mutant TTP

**Figure 10:**
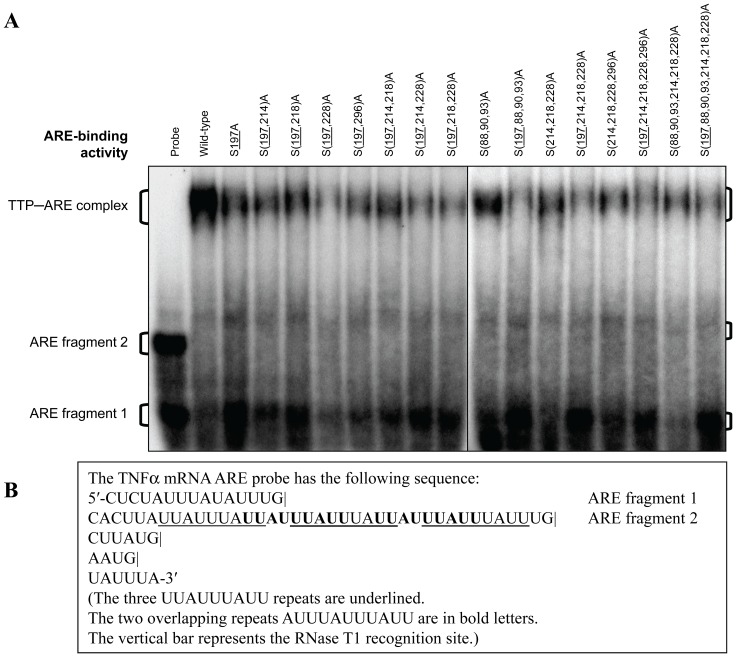
RNA Binding Activity Assay. TNF mRNA ARE binding activity of the wild-type and mutant TTP was evaluated with the RNA gel mobility shift assay. (A) Electrophoretic mobility shift assay. The binding reaction mixtures were incubated for 30 min followed by RNase T_1_ digestion. The TTP−probe complexes and free probes were separated by 6% native PAGE and detected by autoradiography on X-ray film. (B) TNF mRNA ARE probe. The RNA probe was transcribed from mouse TNF mRNA ARE region with the following sequence: 5′-CUCUAUUUAUAUUUG|CACUUAUUAUUUA**UU** **AUUUAUUUAUUAUUUAUUUAUU**UG|CUUAUG|AAUG|UAUUUA-3′ (the three UUAUUUAUU repeats are underlined, and the two overlapping repeats (**UUAUUUAUUUAUUAUUUAUUUAUU**) are in bold letters; the vertical bar represents the RNase T1 recognition site).

RNA gel mobility shift assays were used to evaluate the effect of mutations on TTP’s ability to bind to TNF mRNA ARE. This method showed that both the wild-type and mutant His-hTTP proteins containing one or multiple alanine mutations were all capable of binding to the RNA ARE probes, resulting in accumulation of high molecular size TTP-ARE complexes and disappearance of ARE fragment 2 of the radiolabeled mRNA ARE probes (Figure 10). These results suggest that all mutant proteins tested possess the essential structures for ARE binding under these assay conditions.

## Discussion

TTP is a highly phosphorylated protein in intact cells [1]. Mass spectrometry and site-directed mutagenesis have identified a number of phosphorylation sites in hTTP, including S^66^, S^88^, T^92^, S^169^, S^186^, S^197^, S^218^, S^228^, S^276^ and S^296^, as well as 29 other potential phosphorylation sites [3], [21]. To better understand TTP phosphorylation, we aimed to uncover additional phosphorylation sites by phosphopeptide mapping coupled with *in vivo* labeling, site-directed mutagenesis and mass spectrometry.

The major finding in this report is that the tryptic peptide containing S^90^ and S^93^ is a major phosphopeptide in hTTP. The S90A and S93A mutations in hTTP resulted in the disappearance of a major phosphopeptide with the amino acid sequence LGPELSPSPTSPTATSTTPSR (corresponding to the amino acid residues 83–103 of hTTP) (Figure 9). Mutation at S^197^ resulted in another major phosphopeptide peak being eluted earlier than the wild-type, in agreement with the finding that mutation at this site increases the electrophoretic mobility on SDS-PAGE [21]. Additional mutations at S^186^, S^296^ and T^271^ exhibited little effect on the phosphopeptide mapping profiles. The individual contributions of S^90^ and S^93^ phosphorylation to the overall phosphorylation status of TTP require further investigation. Results from single or double mutations at S^90^ and S^93^ should be able to determine if mutations in these two sites are sufficient for the disappearance of the major radioactivity peak. There is a complete lack of information on the physiological significance of phosphorylation at S^90^ and S^93^ sites in human TTP. Previously, S^93^ was shown to be a potential site for the p38 MAP kinase [3]. The host cells (HEK293 cells) do not express TTP under our culture conditions (Figure 1B) and do not respond to various stimuli. These technical difficulties prevent us from performing experiments to stimulate TTP expression using the constructs in HEK293 cells or other types of cells.

Remarkably, one of the major phosphopeptides was identified in the first C_8_C_5_C_3_H zinc-finger region, and contained three potential phosphorylation sites at T^106^, T^111^ and S^113^ in hTTP. Five potential phosphorylation sites are located in the highly conserved zinc finger domains of hTTP: T^106^, T^111^ and S^113^ in the first zinc-finger region, and Y^158^ and S^160^ in the second zinc-finger region (Figure 9). The biological significance of the potential phosphorylation sites identified in the zinc finger motifs is not clear. The zinc finger domains synthesized chemically or expressed in *E. coli* [5] can bind to the same ARE as the recombinant full-length TTP with similar binding affinity by the electrophoretic mobility shift assay [7]. It is difficult to evaluate the precise effects of phosphorylation at these sites since the binding assay is a semi-quantitative method. It will be important to compare the RNA binding affinity between the phosphorylated and unphosphorylated zinc finger domains in the future.

It is interesting to note that mutation at S^186^ in hTTP (corresponding to S^178^ in mTTP) exhibited little effect on the phosphopeptide mapping profiles. S^186^ in mTTP was shown to be one of the major sites phosphorylated by MK2 *in vivo* and *in vitro* [25]–[28]. MK2 phosphorylation increased TTP protein stability but reduced ARE affinity [33]. The regulation of subcellular localization and protein stability of mTTP is dependent on MK2 and on the integrity of S^52^ and S^178^ [34]. Phosphorylation of mTTP at S^178^ increases the relative ratio of TTP protein in the cytoplasm [32]. Mutation of S^52^ to A^52^ in mTTP weakly reduces the assembly of TTP-14-3-3 protein complex, whereas mutation of S^178^ to A^178^ and of S^52/178^ to A^52/178^ substantially reduces the association of mTTP with 14-3-3 protein complex [35]. Therefore, it will be a great challenge to correlate the phosphorylation sites and the functional consequence in future studies.

It is still difficult to assign the relative contributions of individual phosphorylation sites in TTP. The fact that S90A and S93A mutations in hTTP caused the disappearance of a major phosphopeptide suggests that mass spectrometry alone has limitations on assigning major vs. minor phosphorylation sites. In previous MS analysis, it was suggested that both S^90^ and S^93^ sites in hTTP were minor phosphorylation sites because fewer unique phosphopeptides containing both sites were observed by MudPIT [21]. Instead, S^66^, S^88^, T^92^, S^169^, S^186^, S^197^, S^218^, S^228^, S^276^ and S^296^ were proposed as major sites in hTTP from transfected human cells for a number of reasons [21]: 1) Phosphopeptides containing S^66^, S^88^, T^92^, S^169^ and S^186^ in hTTP observed by MudPIT were confirmed by LC/MS/MS, MALDI/MS/MS or protein sequencing; 2) S^197^, S^218^ and S^228^ strongly affected the electrophoretic mobility of hTTP; 3) More than four copies of phosphopeptides containing S^197^, S^228^, S^276^ and S^296^ were identified from triple digested hTTP by MudPIT; 4) [^32^P]-labeling studies showed that the truncated hTTP peptides containing S^218^ and S^228^ were highly phosphorylated in intact cells; 5) the MALDI MS analysis of the in-gel tryptic digest of hTTP showed ions corresponding in mass to tryptic peptide T20–21/T21–22 (aa 195–242/196–243) plus the addition of one, two, and three phosphate groups; 6) all of these sites were conserved in TTP from various mammalian species; and 7) S^58^, S^176^, S^178^, S^220^, T^250^ and S^264^ of mTTP were phosphorylated *in vivo* and/or *in vitro* [25]; these sites corresponded to S^66^, S^184^, S^186^, S^228^, T^257^ and T^271^ of hTTP. More studies are needed to address the question of relevant contributions of various phosphorylation sites towards the total TTP phosphorylation status in intact cells.

It is thought that phosphorylation of TTP in cells can decrease its mRNA binding activity. For example, TTP expressed in human HEK293 cells and then dephosphorylated by CIAP was able to bind more tightly to an GM-CSF mRNA ARE probe than native, phosphorylated TTP [9]. TTP purified from overexpressed *E. coli* exhibits approximately 2-fold greater affinity for the TNF mRNA ARE than the protein purified from transfected human HEK293 cells [7]. In the present study, wild-type and mutant His-hTTP proteins containing one or multiple phosphorylation site mutations were all capable of binding to ARE-containing RNA probes, resulting in accumulation of high molecular size complexes and disappearance of radiolabeled mRNA ARE probes. These results suggest that the mutant proteins possess the essential structures for ARE binding under these assay conditions. However, quantitative evaluation of TTP-mRNA ARE binding affinity is limited by several factors: 1) It is difficult to normalize the amount of TTP in each protein sample because of other contaminating proteins in the Ni-NTA purified samples; 2) The levels of protein expression are still too low to generate sufficient amounts of purified proteins for quantitative assays; 3) The RNA-gel mobility shift assay is only semi-quantitative, and cannot distinguish minor differences of binding affinity among the TTP forms [7]. Therefore, it is impractical to perform these types of experiments under the current assay conditions. Adaption of other methods after overcoming these technical difficulties is beyond the scope of this study with its focus on identifying major phosphopeptides. Nevertheless, given the potential importance of TTP in cancer progression and inflammation [31], [36], [37], it is important to understand the precise nature and functions of the phosphorylation sites of this important protein in the future.

## Materials and Methods

### Expression Plasmids

The plasmids (pHis-hTTP) coded for recombinant His-hTTP proteins containing six histidine residues at the N-terminus of the full-length hTTP (GenBank accession no. NP_003398) [7], [9]. The site-directed mutant plasmids contained single or multiple alanine mutation(s) at serine and threonine positions in hTTP. The plasmids coding for ten His-TTP proteins were 1) WT, 2) S197A, 3) S(197,228)A, 4) S(197,218,228)A, 5) S(197,214,218,228)A, 6) S(197,214,218,228,296)A, 7) S(88,197,214,218,228,296)A, 8) S(88,186,197,214,218,228,296)A, 9) S(88,197,214,218,228,296)T271A and 10) S(88,90,93,197,214,218,228,296)A [21].

### Transfection of Human HEK293 Cells

HEK293 cells were transiently transfected with pHis-hTTP plasmids using the calcium phosphate precipitation method [7], [12]. HEK293 cells were grown at 37°C with 5% CO_2_ in Dulbecco’s modified Eagle’s medium (DMEM) (Invitrogen) supplemented with 10% (v/v) fetal calf serum (FCS), 100 U/mL penicillin, 100 µg/mL streptomycin, and 2 mM L-glutamine. The cells in 10-cm plates were transfected with 0.5 µg of pHis-hTTP and 4.5 µg of pBS+ carrier plasmids.

### In Vivo Phosphate Radiolabeling

The transfected HEK293 cells were washed after being cultured overnight and incubated in fresh medium under the same conditions for 24 h. The old medium was removed from culture dish followed by washing with no-phosphate DMEM. To the dish was added no-phosphate DMEM plus 1% FCS (approximately 15 µM phosphate in the medium) and incubated at 37°C with 5% CO_2_ for 3 h. The medium was then aspirated off. DMEM (4 mL, no phosphate, no serum) with [^32^P]-orthophosphate (0.1 mCi/mL) was added to each dish [21]. The cells were labeled at 37°C with 5% CO_2_ for 90 min.

### Cell Lysis

The [^32^P]-orthophosphate-medium was removed after *in vivo* radiolabeling. The cells were washed with PBS and lysed directly in the plate at 4°C for 1 h with 0.6 mL His-tag purification buffer (50 mM NaH_2_PO_4_, 250 mM NaCl, 50 mM NaF, 1 mM PMSF, 1 µg/mL leupeptin, 0.5% NP-40) plus 10 mM imidazole. The lysate was transferred into microcentrifuge tubes and centrifuged at 10,000*g* for 10 min. Protein concentrations were determined with the Bio-Rad Dye assay kit (Bio-Rad Laboratories) and BSA as standards [7].

### His-tag Purification

The 10,000 *g* supernatants from transfected HEK293 cells were transferred to 15-mL Falcon tubes and mixed with 5% Ni-NTA beads (Qiagen, Valencia, CA). The mixtures were rotated at 4°C for 2 h and then transferred into a Cytospin column followed by centrifugation at 1000 *g* for 2 min. The beads were washed with wash buffer (50 mM NaH_2_PO_4_, 300 mM NaCl, 50 mM NaF, 0.05% Tween-20, pH 8.0) plus 20 mM imidazole by centrifugation at 1000 *g* for 2 min. The bound proteins were eluted with 100, 200 and 250 mM imidazole in wash buffer by centrifugation at 1000 *g* for 2 min. The eluted proteins and the remaining beads were stored at –20°C.

### Digestion of TTP

His-hTTP proteins eluted from Ni-NTA beads as described above (100 µL) were digested directly for 70 h (extended time for complete digestion) at 37°C with 6 µg of modified trypsin treated with L-(tosylamido-2-phenyl) ethyl chloromethyl ketone (TPCK) (Sigma, St. Louis, MO). The protein was also digested with lysyl endopeptidase (Sigma, St. Louis, MO) under the same conditions.

### HPLC Separation of TTP Peptides

The trypsin-digested peptide mixtures were adjusted to 0.065% TFA by addition of 10% trifluoroacetic acid (TFA). The mixture was set at room temperature for 30 min followed by centrifugation at 10,000 *g* for 10 min. The supernatant was manually injected into a 100 µL loop. The peptide fragments were separated by reverse phase chromatography through a Sephasil Peptide C_18_ 5 µ ST 4.6/100 column (GE Healthcare Life Sciences, Piscataway, NJ) using an AKTA_basic_ system equipped with UNICORN 3.10 version (GE Healthcare Life Sciences, Piscataway, NJ). The peptide mixtures were loaded onto the column from the sample loop with 500 µL of buffer A (0.065% TFA in 2% acetonitrile). The column was washed with 5-bed volumes of buffer A. The peptides were eluted from the column at 0.5 mL/min with a 20-bed volume of a linear gradient from 100% buffer A to 100% of buffer B (0.05% TFA in 100% acetonitrile). The fractions were collected at 0.25 mL/well in 96-well plates with Frac-950 fraction collector (GE Healthcare Life Sciences, Piscataway, NJ). The radioactivity of every fraction was counted using MicroBeta JET (PerkinElmer Life Sciences, Gaithersburg, MD).

### MALDI-MS (matrix-assisted laser-desorption–ionization MS) Analysis

HPLC fractions corresponding to the phosphopeptide peaks from the wild-type protein were analyzed by MALDI MS. The tryptic peptide mixtures were freeze-dried and reconstituted in 5 µL of acetonitrile/water (1∶1, v/v) with 0.1% formic acid. The tryptic peptide solution (0.5 µL) was mixed with 0.5 µL of MALDI matrix consisting of a saturated solution of a-cyano-4-hydroxycinnamic acid in ethanol/water/formic acid (45∶45:10, v/v). MALDI analyses were performed as described [38] using 4700 Proteomics Analyzer (Applied Biosystems, Framingham, MA).

### SDS-polyacrylamide Gel Electrophoresis (PAGE) and Immunoblotting

SDS-PAGE and immunoblotting followed described procedures [39]. The primary antibodies were anti-MBP-hTTP sera raised in New Zealand white rabbits against the purified MBP-TTP fusion protein [17]. The secondary antibodies were affinity-purified goat anti-rabbit IgG (H+L) horseradish peroxidase conjugate with human IgG absorbed (Bio-Rad Laboratories).

### Autoradiography

The undigested His-hTTP proteins and trypsin-digested His-hTTP peptides from radiolabeled cells were separated by SDS-PAGE (Norvex precast, 8–16% Tris-glycine gel). The gel was dried and exposed to X-ray film (Eastman Kodak, Rochester, NY).

### Immunostaining and Confocal Microscopy

HEK293 cells were grown overnight on glass coverslips in tissue culture plate (Becton Dickinson and Company, Lincoln Park, NJ). The cells were transfected with pHis-TTP (50 ng DNA/1 mL/well) and incubated overnight. After another 24-h incubation, the cells were used for immunocytochemistry with anti-MBP-hTTP antibodies using a similar procedure as described [7]. The slides were examined and imaged with an LSM510 UV confocal microscope (Zeiss, Thornwood, NY).

### RNA Binding Activity Assay

TNF mRNA ARE binding activity was evaluated with the RNA gel mobility shift assay (GMSA) according to a previous procedure [7] using a TNF mRNA ARE probe (100000−200000 cpm/reaction) with the sequence shown in Figure 7B. The RNA probe was transcribed from mouse TNF mRNA ARE region (nucleotides 1281−1350 of GenBank accession no. X02611) using [α-^32^P]UTP (NEN Life Sciences, Boston, MA) and T_7_ RNA polymerase with the Promega’s RiboProbe *In Vitro* Transcription System (Promega Corp., Madison, WI). The binding reaction mixtures were incubated for 30 min at room temperature before digestion with 100 units of RNase T_1_ (Epicentre, Madison, WI) for 15 min at 30°C. The TTP−probe complexes and free probes were separated by 6% native PAGE and detected by autoradiography on X-ray film.
